# Preparation of Tung Oil Microcapsule and Its Effect on Wood Surface Coating

**DOI:** 10.3390/polym14081536

**Published:** 2022-04-11

**Authors:** Wenwen Peng, Xiaoxing Yan

**Affiliations:** 1 Co-Innovation Center of Efficient Processing and Utilization of Forest Resources, Nanjing Forestry University, Nanjing 210037, China; yjsww@njfu.edu.cn; 2 College of Furnishings and Industrial Design, Nanjing Forestry University, Nanjing 210037, China

**Keywords:** tung oil microcapsule, performance, self-healing

## Abstract

Through the optimized preparation of tung oil microcapsules, five kinds of microcapsules containing different core material content were obtained to explore the influence of microcapsules on water-based paint film and the self-healing ability of microcapsules. The results showed that the microcapsules had good appearance, and the microcapsules were successfully prepared. The color difference in the paint film increased with the increase in microcapsule content, and the gloss decreased gradually. The mechanical test showed that adding microcapsules increased the toughness of the paint film to a certain extent, and the performance of the paint film was unchanged or better. The results showed that paint film with the core–wall ratio of 0.78:1 had the best performance and self-healing function when microcapsules were added.

## 1. Introduction

The surfaces of wooden furniture are easy to crack, but this can often be prevented by coating, which can also increase the gloss of paint film and beautify furniture [1,2,3]. Water-based paint is the most widely used wood material that saves energy and provides environmental protection [4,5,6]. It has good adhesion and can use water as a solvent in order to save resources. However, water-based paint film is also prone to microcracks, which reduce the service life and increase the repair cost of furniture [7,8,9,10]. Microcapsules make use of the core wall material structure; the repair agent, which functions as the core material of the microcapsule, is wrapped in the wall material, and then the microcapsules are mixed into the water-based paint and coated onto the surface of the furniture [11,12,13]. When the surface paint film cracks and the microcapsule breaks, then the repair agent will flow out to the crack and cure the film at room temperature to repair the crack [14,15,16,17].

Zhang et al. [18] prepared photochromic microcapsules through interfacial polymerization. The microcapsules had good thermal stability, and the factors affecting photochromism were explored. He et al. [19] synthesized photochromic compounds and applied them to cotton fabric. The washing fastness of the fabric was studied, which proved the great potential of the compounds in civil textiles, especially outdoor textiles. Tozum et al. [20] studied the preparation and characterization of several novel thermochromic microcapsules, including surface activity, material safety and the combination of cotton and wool fabrics. Dual-function thermochromic energy storage microcapsules were successfully synthesized, which can be used for reversible discoloration and as a thermal indicator. Xiao et al. [21] successfully prepared microcapsules with NIR light-responsive performance by using near-infrared absorption dye and fluorescent dye as raw materials. The effects of core material and wall material quality, oil phase viscosity and mass ratio of water to oil phase on microcapsule particle size were studied. The results showed that the prepared microcapsules had good color rendering function, the average size of each particle was 0.2–0.5 µm and the imaging accuracy was 20 µm. Feng et al. [22] prepared microcapsules for direct laser writing of polymers and laser-absorbent (SnO_2_) and carbonizable polymers (CP) for core materials and wall materials, respectively. The average size of each particle was 2.2 μm, and the size of the microcapsule’s wall material was 0.21–0.24 μm.

Tung oil is a kind of green vegetable oil with the characteristics of low cost and high temperature resistance; it can be solidified at room temperature. In this study, the mixture of cellulose and urea was the wall material and tung oil was the core material in the original polymerization method used to prepare microcapsules [23,24,25]. By preparing tung oil microcapsules, the self-repair function of paint film on furniture surfaces was demonstrated. When cracks appeared on furniture surfaces, the tung oil from the broken microcapsule flowed to the crack and formed a dense film, using oxidation to repair the cracks. Self-healing microcapsule technology can improve the service life of wooden furniture and reduce the high cost and manpower required to fix paint film cracking.

## 2. Materials and Methods

### 2.1. Experimental Materials

Cellulose (M_w_: 448.47 g/mol, CAS No.: 9004-57-3) was obtained from Jinzhou Honghai Cellulose Technology Co., Ltd., Jinzhou, China. Triethanolamine (M_w_: 149.19 g/mol, CAS No.: 102-71-6) was obtained from Shanghai Beite Chemical Co., Ltd., Shanghai, China. Urea (M_w_: 60.06 g/mol, CAS No.: 57-13-6) was obtained from Nanjing Panfeng Chemical Co., Ltd., Nanjing, China. Citric acid monohydrate (M_w_: 210.14 g/mol, CAS No.: 5949-29-1) was obtained from Shandong Lemon Biochemical Co., Ltd., Anqiu, China. Formaldehyde solution (37%, M_w_: 30.03 g/mol, CAS No.: 50-00-0) was obtained from Jiangsu Changchun Chemical Co., Ltd., Wuxi, China. N-octanol (M_w_: 130.23 g/mol, CAS No.: 111-87-5) and sodium dodecyl benzene sulfonate (SDBS) (M_w_: 348.48 g/mol, CAS No.: 25155-30-0) were obtained from Anyi Chemical Co., Ltd., Nanjing, China. Tung oil was obtained from Guangzhou Chaoya Chemical Co., Ltd., Guangzhou, China. Tung oil contains linolenic acid, linoleic acid, oleic acid and tung oleic acid, all of which have unsaturated double bonds that make tung oil solidify into a film to repair cracks. The unsaturated fatty acids and molecular formula of tung oil are shown in Table 1. Water-based primer, mainly composed of waterborne acrylic acid, copolymer dispersive dimming agent additives and water, has a solid content of about 30.0%. A water-based topcoat, mainly composed of waterborne acrylic acid, polyurethane synthetic additives and water, has a solid content of about 26.5%. The primer and topcoat were obtained from Dulux Co., Ltd., China. Basswood (80 mm × 80 mm × 5 mm) was obtained from Jiangyin Minghe Huzhong Wood Industry Co. Ltd., Wuxi, China.

### 2.2. Preparation of Microcapsules

In order to explore the influence of the quality of core and wall materials on microcapsules, the emulsifier concentration was determined to be 1.0%, the reaction temperature was determined to be 30 °C and the rotation speed was determined to be 900 rpm. Single-factor experiments were conducted by changing the quality of core and wall materials of microcapsules. The required materials and contents are shown in Table 2.

The content of the wall material was kept the same by changing the core–wall ratio and, subsequently, the core material quality. Firstly, the wall material was prepared. Twenty grams of urea and 27.0 g formaldehyde reacted with the volume ratio of 1:1 to generate 27.0 g urea–formaldehyde resin. Urea and formaldehyde were mixed and stirred until the urea was fully dissolved. Then the solution was put into a heating magnetic stirrer, the stirring temperature was adjusted to 70 °C and the stirring speed was adjusted to 600 rpm to react for 60 min. During the reaction, the pH value of the solution was adjusted to 8–9 by adding triethanolamine. Meanwhile, 2.0 g cellulose were mixed with 50 mL water, and the cellulose solution was added and ultrasonically dispersed for 30 min after the urea–formaldehyde resin reaction. Then the core material was prepared, beginning with the emulsifier solution. SDBS was used as an emulsifier and mixed with water, and then the tung oil was added into the magnetic stirrer. The reaction temperature was adjusted to 45 °C, and the stirring rate was 1000 rpm for 30 min. Then the core liquid and the wall liquid were mixed, the temperature of the magnetic mixer was adjusted to 30 °C and the stirring speed was adjusted to 900 rpm. After the solution was fully mixed, citric acid monohydrate solution was dropped to change the pH to acidic for 3–4 h; the solution then reacted for 2 h. After that, the mixture was left for 5 d; then we filtered, washed and dried the mixture to obtain microcapsules of white powder.

The urea–formaldehyde resin in the microcapsule wall material was configured in accordance with the volume ratio of 1:1, which ensured that the formaldehyde was completely reacted. Cellulose and tung oil are natural environmental protection materials and do not have toxicity. Therefore, the microcapsules prepared were non-toxic.

### 2.3. Preparation of Coating

In order to study the effect of microcapsules on paint film, the waterborne paint with microcapsules was prepared by adding microcapsules for the primer and topcoat at the same time. The microcapsules were added to primer and brushed three times. Then the microcapsules were added to the topcoat and brushed three times. The microcapsule content was divided into two equal parts, which were added to the primer and topcoat, respectively, then mixed evenly and coated onto the wood surface. The effect of microcapsule content on the surface properties of paint film was studied by changing the content of the microcapsules. The thickness of the paint film was 25 mm × 100 mm and the weight was 0.12–0.20 g. Five different concentrations were set for this purpose, and the ratio of water-based paint with tung oil microcapsules is shown in Table 3.

### 2.4. Testing and Characterization

According to the GB/T 6739-2006 paint and varnish standard, we used the pencil method to determine the hardness of the paint film. The hardness of the paint film was tested by a 298 pencil hardness tester, which was obtained from Shanghai Litao Automation Technology Co., Ltd., Shanghai, China. The hardness pencil was placed on a mechanical car at a 45° angle, exerting downward pressure on the surface of the paint film. Then, by forcing the car to drive evenly, the pencil left a scratch on the surface of the paint film. The scratch results were observed with a magnifying glass, and the greater the pencil H value, the stronger the hardness.

According to Part 4 of the standard GB/T 4893.4 2013 physical and chemical properties test of paint film on furniture surfaces, we used the adhesion cross cutting method to test the adhesion of the paint film. The adhesion was tested by a QFH film scriber, which was obtained from Hebei Zhongke Beigong Test Instrument Co., Ltd., Cangzhou, China. The surface of the wood was cut with multiple blades, then the wood was rotated 90 degrees to make the final cut into a grid pattern. The tape was pasted on the grid graph, and then torn off smoothly within 0.5–1.0 s, so the results could be observed on the tape. Adhesion grade was determined as shown in Table 4.

According to the impact resistance test described in Part 9 of the standard GB/T 4893.9 2013 physical and chemical properties test of paint film on furniture surfaces, the impact resistance of paint film was tested by a QCJ-120 impact testing machine, which was obtained from Shenzhen Sanuo Instrument Co., Ltd., Shenzhen, China. A wood test board coated with paint film was placed on the horizontal base of the impactor and fixed. The impact ball was raised to a certain height, and the switch was pressed to let the ball fall free to impact the plate. Then it was observed whether there were impact marks and cracks near the impact of the ball, and the height of the ball when the crack occurred was recorded.

The elongation at the break of the paint film was tested by an MTest-i universal mechanical testing machine which was obtained from Shanghai Yinhuang Technology Co., Ltd., Shanghai, China. The paint film was coated on the glass plate in accordance with the coating process, then removed from the glass. Then we used the universal mechanical testing machine to test elongation at the break of the paint film according to the Formula (1). L_0_ represents the original length of the paint film, L represents the length of the paint film at the time of the break and ***e*** represents elongation at the break of the paint film.

(1) $$e=\frac{\left(L-L_{0}\right)}{L_{0}}\times 100\%$$

A tiny crack in the paint film was cut with a razor blade and then observed under a microscope. After five days in the paint film, the same spot in the crack was observed with an electron microscope. Repair rate (R) was calculated according to the Formula (2), where L_1_ represents the crack width before repair and L_2_ represents the crack width after repair.

(2) $$R=\frac{\left(L_{1}-L_{2}\right)}{L_{1}}\times 100\%$$

The color difference of paint film was tested by a TS8260 portable color meter, which was obtained from Suzhou Weifu Photoelectric Technology Co., Ltd., Suzhou, China. By testing two places on the paint film, L_1_, a_1_, b_1_ and L_2_, a_2_, b_2_ were tested. The color difference ΔE of the paint film was calculated by Formula (3).

(3) $$\Delta E={\left[{\left(\Delta L\right)}^{2}+{\left(\Delta a\right)}^{2}+{\left(\Delta b\right)}^{2}\right]}^{1/2}$$

The gloss of paint film was tested by an LS195 glossometer, which was obtained from Shenzhen Linshang Technology Co., Ltd., Shenzhen, China. The roughness of paint film was tested by a Jb-4c precision roughness tester, which was obtained from Shanghai Taiming Optical Instrument Co., Ltd., Shanghai, China. The smoother the film surface, the smaller the value. The liquid resistance of paint film was tested by distilled water, citric acid (Shandong Lemon Biochemical Co., Ltd., Weifang, China), detergent (LIbY Group Co., Ltd., Guangzhou, China) and disinfectant (Taizhou Changcheng Detergent Factory Co., Ltd., Taizhou, China). The filter paper of the same size was first immersed in four kinds of solutions, and then placed on the surface of the paint film. The test sample was covered with glass and left for 24 h, then the filter paper was removed and the residual liquid on the surface of the paint film was wiped with a cloth. The marks and changes in the test area and the liquid resistance level were observed. The liquid resistance level of the paint film is shown in the Table 5.

The microstructure and chemical constitution of the paint film were observed by optical microscopy (OM) (Leica Microsystems Co., Ltd., Weztlar, Germany), field emission scanning electron microscopy (SEM) (TESCAN Co., Ltd., Brno, Czech Republic) and Nicolet iS5 Fourier transform infrared spectroscopy (FTIR) (Thermo Fisher Scientific Co., Ltd., Waltham, MA, USA).

## 3. Results and Discussion

### 3.1. Microcapsule Microanalysis

**Figure 1:**
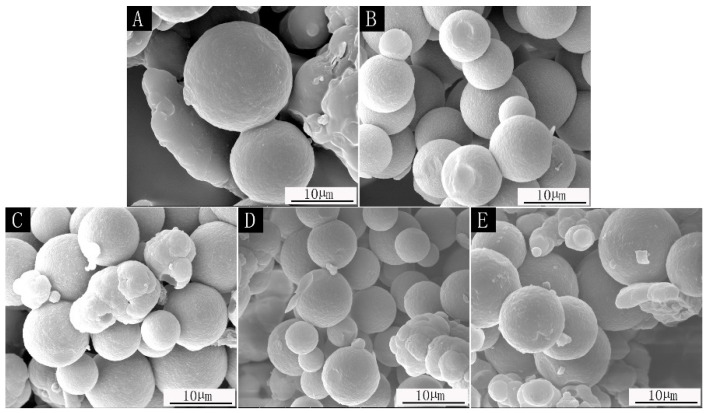
SEM of microcapsules: (**A**) core–wall ratio is 0.42:1; (**B**) core–wall ratio is 0.54:1; (**C**) core–wall ratio is 0.65:1; (**D**) core–wall ratio is 0.78:1; (**E**) core–wall ratio is 0.89:1.

**Figure 2:**
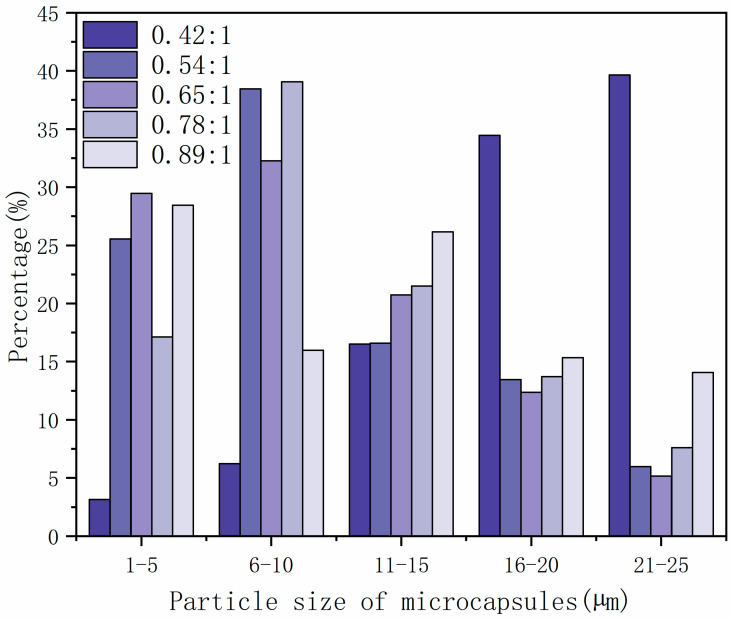
The particle size distribution of microcapsules with different core–wall ratios.

SEM images of microcapsules prepared with different core material content are shown in Figure 1, and the particle size distribution of microcapsules with different core–wall ratios is shown in Figure 2. The prepared microcapsules were round and the particle sizes were basically similar. The forming microcapsules with a 0.42:1 core–wall ratio were fewer and the particle size was larger, which is probably because the core material was too low in the emulsion system. The 0.65:1 and 0.89:1 microcapsules showed agglomeration phenomena, while the 0.54:1 and 0.78:1 microcapsules showed better morphology with less agglomeration.

**Figure 3:**
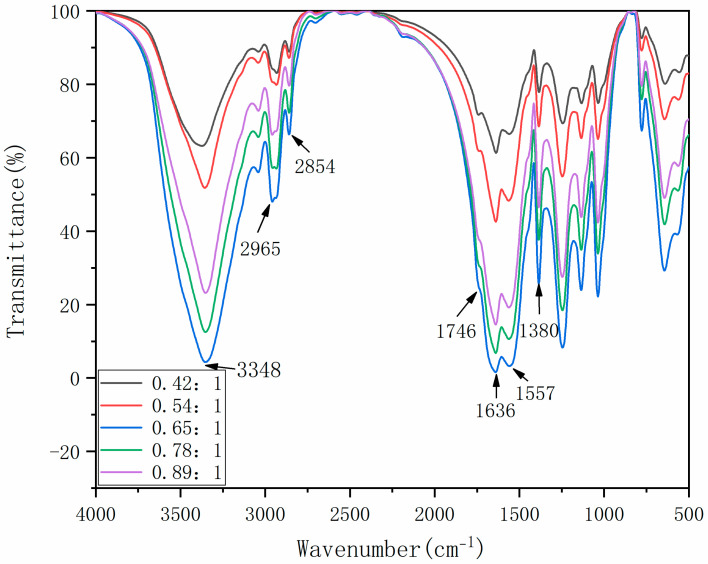
FTIR of microcapsules.

The FTIR analysis results of microcapsules are shown in Figure 3. The stretching vibration peaks of N-H and O-H are superimposed at 3348 cm^−1^, and the stretching vibration peaks of C=O and C-N at 1636 cm^−1^ and 1557 cm^−1^, respectively. The absorption bimodal peaks near 2965 cm^−1^ are from the stretching vibration of methylene in cellulose molecular structure, and the characteristic absorption peak at 1380 cm^−1^ represents the stretching vibration of the methyl group. These are the characteristic peaks of wall material. The stretching vibration peak of C-H at 2854 cm^−^^1^ and the stretching vibration peak of C=O at 1746 cm^−1^ are all characteristic peaks of tung oil in the core material. The presence of core material and wall material in the whole spectrum shows that the microcapsule coating was successful.

### 3.2. Mechanical Property Test

The viscosity of the water paint is shown in Table 6. The viscosity of the water paint increased with the increase in microcapsule content. When the microcapsule content reached 12.0%, the viscosity of the film reached 39 s. The core–wall ratio had little effect on the viscosity of waterborne paint because the microcapsules prepared were powder.

By changing the content of microcapsules, this study explored the mechanical properties of microcapsules on paint film. Table 7 shows the test results of paint film hardness. With the continuous increase in microcapsule content, the hardness of the paint film also continued to increase by small amounts, indicating that the microcapsules could increase the original hardness of the paint film up to a point. This was mainly because the microcapsules themselves were tiny particles, and the cellulose increased the toughness of the paint film up to a point, thus increasing the hardness of the paint film [26,27]. The core–wall ratios of microcapsules were 0.54:1, 0.78:1 and 0.89:1, which had better enhancement influence on the hardness.

**Figure 4:**
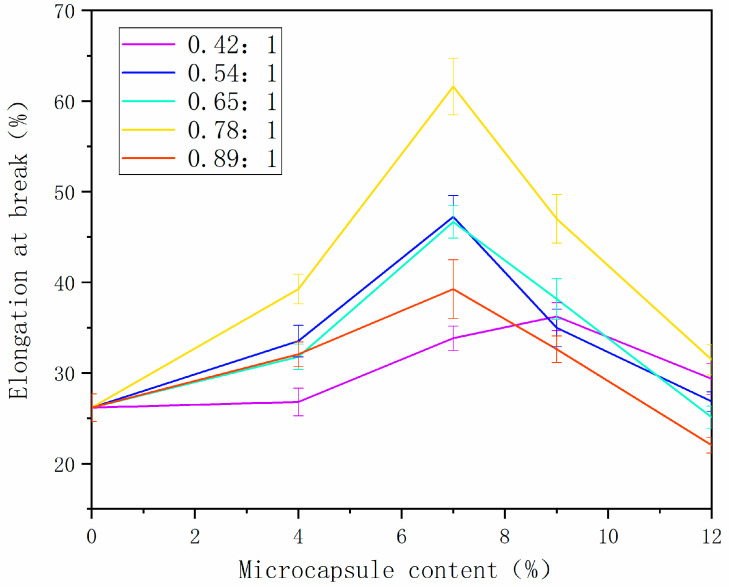
Change in breaking elongation of paint film prepared with different contents of tung oil.

Figure 4 shows the analysis of the breaking elongation of the paint film. With the increase in microcapsule content, the breaking elongation of paint film with different core–wall ratios increased first and then decreased. This was because the cellulose increased toughness to some extent, and, when added, the microcapsules improved the breaking elongation of paint film [28,29]. Due to too much microcapsule powder, the adhesive was not enough to completely wrap the powder, and the binding force between the powder and the binder was poor, which reduced the performance. The elongation at break of paint film increased from 26.26% to 60.65% when the 0.78:1 microcapsules were added. Microcapsules have two functions: one is toughening, making the coating less prone to cracking, and the other is self-healing. If cracks occur, the cracks can be repaired. Elongation at break is a mechanical property of the coating, due to the toughness of the coating. The greater the elongation at break, the better the toughness of the coating, and the greater the increase in the service life of the coating.

Adhesion demonstrates the mutual attraction between wood and paint film, and adhesion grade determines whether paint film and wood surface can be a good joint, making wood and air isolation. The lower the adhesion grade, the smoother the cutting edge and the better the adhesion. The adhesion grades are shown in Table 8. The adhesion grade of the paint film without adding microcapsules was 0, and this adhesion was the best. When the core–wall ratios were 0.54:1 and 0.78:1 and the microcapsule content was 0–7%, the adhesion grade of the film was 0 and the adhesion was the best. With the increase in microcapsule content from 0% to 12%, the adhesion grade of the paint film rose from 0 grade to 2–3 grade, and the adhesion became worse. Water-based paint without microcapsules had no impurities and could be better cured into a film. When the microcapsules were added, the microcapsules were dispersed in the waterborne paint, affecting the adhesion and the interface bonding capability of the paint film. According to the overall analysis, the adhesion of the water-based paint film with 0.54:1 and 0.78:1 microcapsules was less affected by the content of the microcapsules, and the adhesion of the water-based paint film was relatively better.

The research results of the impact resistance of the water-based paint film containing microcapsules are in Table 9. When the content of microcapsules increased from 0% to 12.0%, the impact resistance of the paint film increased gradually. This was mainly because granular microcapsules increased the hardness to a certain extent, increasing the impact resistance as well [30]. The impact resistance of the 0.54:1, 0.78:1 and 0.89:1 microcapsules increased with the increase in microcapsule content, which increased from the original 10 kg·cm to about 20 kg·cm.

Roughness values of paint film are shown in Table 10. By exploring the roughness of paint film, it is clear that the higher the content of microcapsules, the greater the roughness of paint film. The main reason is that when the content of microcapsules increases, the microcapsules cannot mix well with water-based paint, leading to the agglomeration of microcapsules and the increasing roughness of the paint film. Under different core–wall ratios, the roughness values of the paint film with the same microcapsule content show little difference.

### 3.3. Optical Property Test

**Figure 5:**
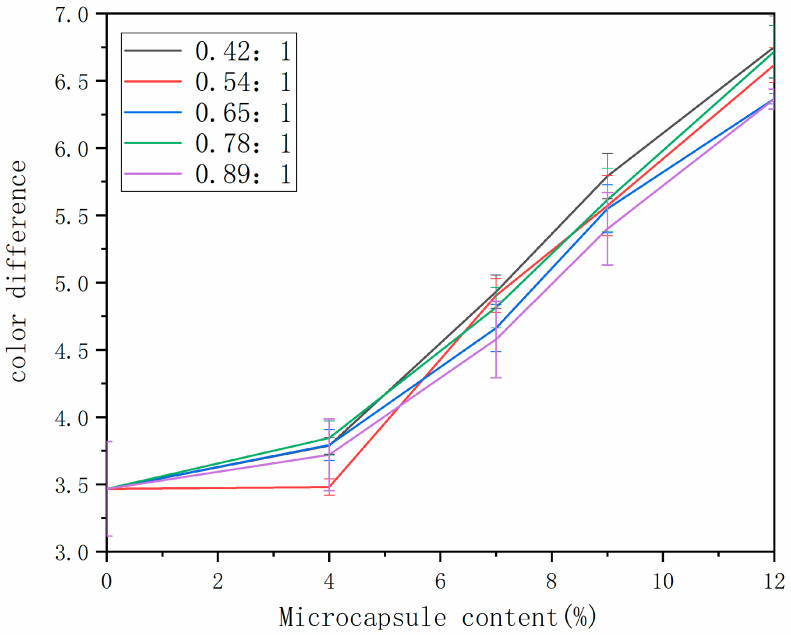
Change in color difference of paint film prepared with different contents of tung oil.

Through the study of the optical properties of the water-based paint film, the changes in color difference and gloss on the surfaces of wooden furniture before and after the addition of microcapsules were explored to judge the impact of microcapsule content on the appearance of the water-based paint film. The change in paint film color difference is in Figure 5. The color difference of the paint film increased with the increase in microcapsule content, and the color difference changed slowly when the microcapsule content was small. This was mainly because the microcapsules were white powder, and the color difference in the water-based paint film without microcapsules was the original color difference of the wood surfaces [31,32]. When the white microcapsules were added to the water-based paint, the original color of the wood was affected, resulting in the increasing color difference of the wood surface paint film. Different core–wall ratios had less effect on the color difference in paint film.

The results of the study on the gloss are shown in Table 11. When more and more microcapsules were added, the gloss of the paint film gradually decreased. This was because, when the content of the microcapsules was greater, the surface of the paint film was rough and uneven, which resulted in diffuse reflection of light and led to the gloss decreasing [33,34]. The core–wall ratio of microcapsules was different, and the gloss was different. Among them, the gloss of the microcapsule core–wall ratios of 0.54:1 and 0.78:1 decreased more slowly.

### 3.4. Liquid Resistance Test

With the increase in microcapsule content, the rheological properties of the coating changed, thus affecting the mechanical and optical properties of the coating [35,36,37]. According to the above study on the mechanical and optical performances of the paint film with different core–wall ratio microcapsules, it can be concluded that the paint film with 0.54:1 and 0.78:1 microcapsules had better performance. In view of the liquid resistance of the paint film, four different liquids were selected to explore the paint film resistance of microcapsules with two kinds of core material content. Then the hardness, adhesion and impact resistance of the liquid-resistant film were tested to explore the mechanical properties of the liquid-resistant film. The results are shown in Table 12. The antiseptic properties of the paint film are all poor, and obvious bubbling and discoloration can be seen after liquid resistance. The paint film’s water resistance, acid resistance and detergent resistance were similar. The paint film with 0.78:1 microcapsules had better resistance to citric acid and detergent, which was grade 1.

After the liquid resistance test, the mechanical properties of the paint film after liquid resistance were measured, and the results are shown in Table 13. Test 1 represents hardness, Test 2 represents adhesion and Test 3 represents impact resistance. The hardness of the paint film after liquid resistance without microcapsules was worse than that with microcapsules. This was mainly because of the cellulose in the wall material of microcapsules, which increased the toughness to a certain extent, thus enhancing the adhesion [38,39]. The hardness of paint film after disinfectant resistance was lower than other hardness, because the grade of paint film disinfectant resistance was poor, so the hardness of liquid resistance was poor too. The adhesion of the water-based paint film after water and acid resistance was basically unchanged, but the adhesion of the water-based paint film after disinfectant and detergent resistance was increased to a certain extent, showing that the effects of disinfectant and detergent resistance were poor. In general, the resistance property of the paint film with 0.78:1 microcapsules was better than that of the paint film with 0.54:1 microcapsules.

### 3.5. Self-Healing Property Test

**Figure 6:**
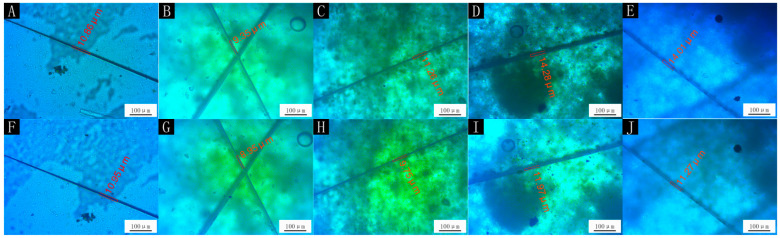
OM of self-healing test of paint film with 0.78:1 microcapsule: (**A**) before healing, with 0%; (**B**) before healing, with 4.0%; (**C**) before healing, with 7.0%; (**D**) before healing, with 9.0%; (**E**) before healing, with 12.0%; (**F**) after healing, with 0%; (**G**) after healing, with 4.0%; (**H**) after healing, with 7.0%; (**I**) after healing, with 9.0%; (**J**) after healing, with 12.0%.

In accordance with the optical, mechanical and fluid resistance properties of the microcapsules we tested, the properties of the paint film with 0.78:1 microcapsules were the best. Therefore, self-repair experiments were carried out on the paint film to explore whether the microcapsules had the effect of repairing cracks. Figure 6 shows the effects of different amounts of microcapsules on the self-healing performance of the paint film. The picture in the first row corresponds to the picture in the second row, which represents the same paint film on different days. The picture in the first row shows the paint film after one day and the picture in the second row shows the film after five days. The paint film without microcapsules had artificial scratches and was left for five days after the split of the paint film, by which time the crack had grown. The paint film containing microcapsules had artificial scratches and was left for five days after the split of the paint film, by which time the crack was narrow. While the repair rate of the paint film with 4.0% microcapsules was only 4.27%, the repair rate of the paint film with 7.0% microcapsules was 13.41%, the repair rate with 9.0% microcapsules was 16.17% and the repair rate with 12.0% microcapsules was 19.56%. With the increased number of microcapsules, the paint film crack was repaired more obviously. This was because the increased content of microcapsules led to more repair-agent core material, which repaired the cracks better. For reference [40], the repair rate of the self-healing coating was 39.0%, which was better than the current repair rate. However, tung oil microcapsules also have certain self-healing properties and have better hardness, adhesion, and impact resistance than shellac waterborne coating microcapsules.

**Figure 7:**
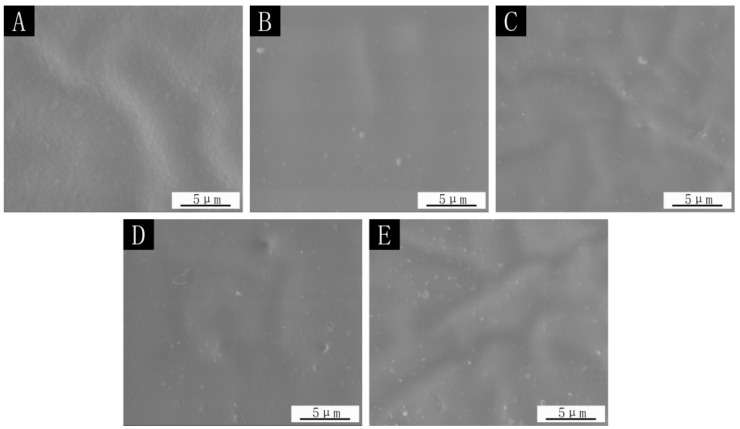
SEM images of 0.78:1 microcapsule paint film: (**A**) 0%, (**B**) 4.0%, (**C**) 7.0%, (**D**) 9.0%, (**E**) 12.0%.

**Figure 8:**
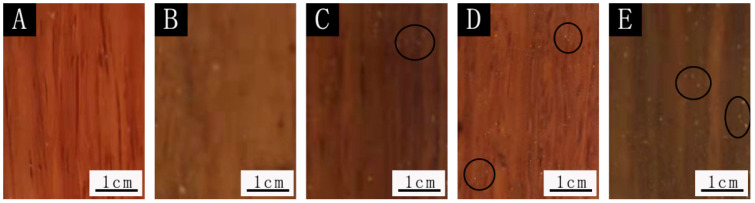
Optical photographs of the coating with 0.78:1 microcapsule paint film: (**A**) 0%, (**B**) 4.0%, (**C**) 7.0%, (**D**) 9.0%, (**E**) 12.0%.

The SEM images and optical photographs with the 0.78:1 microcapsules in the film are shown in Figure 7 and Figure 8. It can be seen that the surface of the film without microcapsules was smooth and there was no foreign matter. When microcapsules were added, the particles (the circled places in Figure 8) appeared on the surface of the paint film, resulting in an increase in the roughness value of the paint film. The more microcapsules were added, the more particles appeared on the surface of the paint film, and the surface became rougher.

## 4. Conclusions

It can be concluded that the core material and wall material constituted microcapsules. When the number of microcapsules increased, the color difference, hardness and impact resistance of the paint film gradually increased, and the gloss gradually decreased. The adhesion grade became higher, which meant that the adhesion of the paint film descended with the rise of microcapsules. The elongation at the break in the paint film rose first and then descended. After comprehensive analysis, the comprehensive performance of 0.78:1 microcapsules was better. The results show that microcapsules had certain self-healing properties, and the higher the content of microcapsules, the better the self-healing properties of paint film. The microcapsules can enhance the performance of paint film to a certain extent and repair the tiny cracks, which increases the service life of furniture-surface paint film and reduces repair costs, providing technical support for the further study of the application of microcapsules in furniture-surface paint film in the future.

## Figures and Tables

**Table 1:** Unsaturated fatty acids and molecular formula of tung oil.

Unsaturated Fatty Acid	Molecular Formula
Linolenic acid	HOOC(CH_2_)_7_CH=CHCH_2_CH=CHCH_2_CH=CHCH_2_CH_3_
Linoleic acid	HOOC(CH_2_)_7_CH=CHCH_2_CH=CH(CH_2_)_4_CH_3_
Oleic acid	HOOC(CH_2_)_7_CH=CH(CH_2_)_7_CH_3_
Eleostearic acid	HOOC(CH_2_)_7_CH=CHCH=CHCH=CH(CH_2_)_3_CH_3_

**Table 2:** Experimental materials table.

Core–Wall Ratio	Urea (g)	Formaldehyde (g)	Cellulose (g)	Tung Oil (g)	SDBS (g)	Deionized Water (mL)
0.42:1	20.0	27.0	2.0	13.44	1.34	132.66
0.54:1	20.0	27.0	2.0	17.28	1.73	171.27
0.65:1	20.0	27.0	2.0	20.80	2.08	205.92
0.78:1	20.0	27.0	2.0	24.96	2.50	247.50
0.89:1	20.0	27.0	2.0	28.48	2.85	282.15

**Table 3:** Water-based paint ratio table with microcapsules added.

Proportion of Microcapsules (%)	Water-Based Paint Content (g)	Microcapsule Content (g)	Total Content (g)
0	18.00	0.00	18.00
4	17.28	0.72	18.00
7	16.74	1.26	18.00
9	16.38	1.62	18.00
12	15.84	2.16	18.00

**Table 4:** Determination of adhesion of paint film.

Adhesion Grade	Illustrate
1	The edges and corners of the grid do not fall off the paint film
2	The edge of the grid has obvious paint film peeling
3	The edges and corners of the grid have obvious paint film peeling and the peeling area is greater than 15% and less than 35%
4	The edges and corners of the grid have obvious paint film peeling and the peeling area is greater than 35% and less than 65%

**Table 5:** Determination of liquid resistance level of paint film.

Resistance to Liquid Level	Change in Paint Film
1	Print-free
2	Slight discoloration
3	Slight discoloration or marked discoloration
4	Marked changes, blistering, wrinkles, etc.

**Table 6:** Change of viscosity of paint film prepared with different contents of tung oil.

Core–Wall Ratio	Viscosity (s)				
	0%	4%	7%	9%	12%
0.42:1	35	35	36	38	38
0.54:1	35	35	36	37	38
0.65:1	35	36	36	38	39
0.78:1	35	35	37	38	39
0.89:1	35	35	36	37	38

**Table 7:** Change of hardness of paint film prepared with different contents of tung oil.

Core–Wall Ratio	Hardness				
	0%	4%	7%	9%	12%
0.42:1	3H	3H	4H	4H	5H
0.54:1	3H	4H	4H	5H	6H
0.65:1	3H	3H	4H	5H	5H
0.78:1	3H	4H	5H	5H	6H
0.89:1	3H	4H	4H	5H	6H

**Table 8:** Change in adhesion of paint film prepared with different contents of tung oil.

Core–Wall Ratio	Adhesion (Grade)				
	0%	4%	7%	9%	12%
0.42:1	0	1	1	2	2
0.54:1	0	0	0	1	2
0.65:1	0	1	1	2	3
0.78:1	0	0	0	1	2
0.89:1	0	0	1	2	3

**Table 9:** Change in impact resistance of paint film prepared with different contents of tung oil.

Core–Wall Ratio	Impact Resistance (kg·cm)				
	0%	4%	7%	9%	12%
0.42:1	10	12	15	17	17
0.54:1	10	13	17	20	21
0.65:1	10	10	12	16	17
0.78:1	10	12	16	22	23
0.89:1	10	11	14	19	22

**Table 10:** Change in roughness of paint film prepared with different contents of tung oil.

Core–Wall Ratio	Roughness (μm)				
	0%	4%	7%	9%	12%
0.42:1	0.36 ± 0.01	0.40 ± 0.01	0.52 ± 0.02	0.57 ± 0.01	0.66 ± 0.01
0.54:1	0.36 ± 0.01	0.41 ± 0.01	0.49 ± 0.01	0.59 ± 0.02	0.66 ± 0.02
0.65:1	0.36 ± 0.01	0.38 ± 0.01	0.46 ± 0.01	0.53 ± 0.02	0.68 ± 0.01
0.78:1	0.36 ± 0.01	0.38 ± 0.01	0.47 ± 0.02	0.50 ± 0.02	0.63 ± 0.01
0.89:1	0.36 ± 0.01	0.47 ± 0.02	0.58 ± 0.01	0.63 ± 0.01	0.76 ± 0.02

**Table 11:** Change in gloss of paint film prepared with different weight of tung oil.

Core–Wall Ratio	Microcapsule Content (%)	20° (%)	60° (%)	85° (%)
0.42:1	0	52.9 ± 1.17	78.5 ± 1.13	89.6 ± 1.68
	4	31.7 ± 1.90	59.1 ± 1.79	58.3 ± 1.78
	7	27.5 ± 0.67	45.0 ± 1.93	55.6 ± 1.34
	9	14.0 ± 0.78	33.4 ± 1.09	49.7 ± 1.44
	12	12.9 ± 0.31	25.8 ± 0.30	29.9 ± 0.73
0.54:1	0	52.9 ± 1.17	78.5 ± 1.13	89.6 ± 1.68
	4	27.4 ± 0.98	61.1 ± 1.11	66.9 ± 1.22
	7	18.6 ± 0.30	55.8 ± 1.45	57.8 ± 1.23
	9	16.8 ± 1.68	47.6 ± 1.59	41.5 ± 0.65
	12	15.7 ± 1.75	49.1 ± 1.58	51.4 ± 1.11
0.65:1	0	52.9 ± 1.17	78.5 ± 1.13	89.6 ± 1.68
	4	37.9 ± 1.07	52.1 ± 0.82	63.4 ± 1.60
	7	22.7 ± 0.66	37.8 ± 1.18	47.6 ± 3.31
	9	17.6 ± 0.95	30.9 ± 0.72	41.5 ± 4.18
	12	11.3 ± 0.68	16.7 ± 1.12	17.5 ± 0.37
0.78:1	0	52.9 ± 1.17	78.5 ± 1.13	89.6 ± 1.68
	4	27.0 ± 0.76	66.5 ± 0.71	77.5 ± 0.21
	7	21.9 ± 1.84	51.9 ± 0.57	58.4 ± 1.28
	9	25.5 ± 1.79	55.6 ± 1.72	58.2 ± 1.32
	12	16.4 ± 1.21	49.8 ± 1.51	48.8 ± 1.16
0.89:1	0	52.9 ± 1.17	78.5 ± 1.13	89.6 ± 1.68
	4	24.5 ± 1.08	54.1 ± 1.88	49.2 ± 0.99
	7	14.5 ± 1.46	31.2 ± 0.95	29.4 ± 0.99
	9	11.6 ± 1.07	22.2 ± 1.58	21.7 ± 0.54
	12	9.6 ± 0.29	19.9 ± 0.94	13.8 ± 0.43

**Table 12:** Resistance grade of water-based paint film.

Core–Wall Ratio	Microcapsule Content (%)	Resistance to Liquid Level			
		Distilled Water	Citric Acid	Disinfectant	Detergent
0.54:1	0	1	1	4	1
	4	1	1	3	1
	7	1	1	3	2
	9	1	1	2	2
	12	1	2	3	1
0.78:1	0	1	1	4	1
	4	2	1	3	1
	7	1	1	2	1
	9	1	1	2	1
	12	1	1	3	1

**Table 13:** Mechanical properties of paint film after resistance to liquid.

Core–Wall Ratio	Microcapsule Content (%)	Distilled Water			Citric Acid			Disinfectant			Detergent		
		Test 1	Test 2	Test 3	Test 1	Test 2	Test 3	Test 1	Test 2	Test 3	Test 1	Test 2	Test 3
0.54:1	0	3 H	0	11	3 H	1	12	2 H	1	14	3 H	0	13
	4	5 H	1	12	5 H	1	13	3 H	3	12	4 H	2	12
	7	4 H	1	12	5 H	1	12	3 H	3	11	4 H	1	12
	9	5 H	1	12	5 H	1	12	3 H	3	14	4 H	2	12
	12	5 H	1	12	4 H	1	13	4 H	2	13	5 H	1	12
0.78:1	0	3 H	0	11	3 H	1	12	2 H	1	14	3 H	0	13
	4	5 H	1	13	4 H	1	13	3 H	2	14	5 H	4	13
	7	4 H	1	15	5 H	1	13	3 H	2	14	5 H	3	13
	9	5 H	1	13	5 H	1	13	3 H	2	15	5 H	2	14
	12	5 H	1	13	5 H	1	12	4 H	3	14	4 H	1	13

## Data Availability

Not applicable.
